# Hospital doctors’ attitudes to brief educational messages that aim to modify diagnostic test requests: a qualitative study

**DOI:** 10.1186/s12911-020-1087-2

**Published:** 2020-04-29

**Authors:** Ben Young, Andrew W. Fogarty, Rob Skelly, Dominick Shaw, Nigel Sturrock, Mark Norwood, Peter Thurley, Sarah Lewis, Tessa Langley, Jo Cranwell

**Affiliations:** 1 Division of Epidemiology and Public Health, Clinical Sciences Building, University of Nottingham, Nottingham City Hospital, Hucknall Road, Nottingham, NG5 1PB UK; 2 Royal Derby Hospital, Uttoxeter Road, Derby, DE22 3NE UK; 3 NIHR Nottingham Biomedical Research Centre, Clinical Sciences Building, University of Nottingham, Nottingham City Hospital, Hucknall Road, Nottingham, NG5 1PB UK; 4 Department for Health, University of Bath, Claverton Down, Bath, BA2 7AY UK

**Keywords:** Unnecessary testing, Computed tomography, Behavioural economics, Doctor decision-making, Intervention acceptability

## Abstract

**Background:**

Avoidable use of diagnostic tests can both harm patients and increase the cost of healthcare. Nudge-type educational interventions have potential to modify clinician behaviour while respecting clinical autonomy and responsibility, but there is little evidence how this approach may be best used in a healthcare setting. This study aims to explore attitudes of hospital doctors to two nudge-type messages: one concerning potential future cancer risk after receiving a CT scan, another about the financial costs of blood tests.

**Methods:**

We added two brief educational messages to diagnostic test results in a UK hospital for one year. One message on the associated long-term potential cancer risk from ionising radiation imaging to CT scan reports, and a second on the financial costs incurred to common blood test results. We conducted a qualitative study involving telephone interviews with doctors working at the hospital to identify themes explaining their response to the intervention.

**Results:**

Twenty eight doctors were interviewed. Themes showed doctors found the intervention to be highly acceptable, as the group had a high awareness of the need to prevent harm and optimise use of finite resources, and most found the nudge-type approach to be inoffensive and harmless. However, the messages were not seen as personally relevant because doctors felt they were already relatively conservative in their use of tests.

Cancer risk was important in decision-making but was not considered to represent new knowledge to doctors. Conversely, financial costs were considered to be novel information that was unimportant in decision-making. Defensive medicine was commonly cited as a barrier to individual behaviour change. The educational cancer risk message on CT scan reports increased doctors’ confidence to challenge decisions and explain risks to patients and there were some modifications in clinical practice prompted by the financial cost message.

**Conclusion:**

The nudge-type approach to target avoidable use of tests was acceptable to hospital doctors but there were barriers to behaviour change. There was evidence doctors perceived this cheap and light-touch method can contribute to culture change and form a foundation for more comprehensive educational efforts to modify behaviour in a healthcare environment.

## Background

There is growing awareness internationally that medical services are under pressure due to a variety of factors, including the overuse of the diagnostic tests and therapies available [1]. One example of this phenomenon is in medical imaging, as computed tomography (CT) scans have increased by 69% in absolute numbers since 2012–13 in England, with approximately 5.67 million CT scans performed by the National Health Service (NHS) in 2018–19 [2]. While many CT scans are undoubtedly important in detecting treatable disease, some of this increase in demand may reflect the increased availability of CT scans, despite the fact that they are well recognised to increase the lifetime risk of cancer [3]. Other diagnostic tests that may be overused include pathology tests such as blood tests, resulting in psychological and social impact to patients [4]. In the context of a UK NHS that is extremely financially overstretched due to a funding shortfall [5], the routine use of 1.1 billion pathology tests per year costs £2.2 billion [6], and any intervention that can promote more rational use of these tests may improve quality and efficiency of health care delivery.

The proportion of medical tests estimated to be unnecessary by a sample of 2106 American Medical Association doctors was 25% [7]. Another study found 59% of 824 hospital doctors in Pennsylvania, USA reported that they often order more tests than medically indicated [8]. Overuse of tests is difficult to measure objectively but unexpected variations in recorded usage can indicate probable overuse [1]. Variations observed include an approximately threefold increase in the rate of use of CT scans over the period of transition from paediatric to adult care [9], differences in spending between NHS acute hospitals [10] and different thresholds between doctors for ordering diagnostic tests [11, 12]. Changing individual and organisational behaviour is fundamental to achieving a reduction in overuse of routine diagnostic tests by hospital doctors.

Best approaches to improve doctors’ use of diagnostic tests have not yet been established, and are likely to involve a number of complementary approaches to convey educational messages to influence decisions about whether or not to order tests. A systematic review of interventions to reduce unnecessary paediatric imaging and pathology testing found that interventions were more effective if they targeted either imaging or pathology testing rather than both simultaneously [13]. Behavioural science provides awareness of the cognitive processes that underpin decision-making and can guide the development of interventions with a view to influencing clinician decision-making in a busy hospital environment. ‘Nudge-type’ approaches operate mainly through subconscious automatic, rather than reflective, processes, to influence decisions while maintaining the autonomy of the decision-maker, and are easy and cheap to avoid [14]. They have been used with varying results in health care to reduce antibiotic prescribing [15, 16], promote handwashing [17] and reduce clinician orders for ‘low-value’ services [18]. A very light-touch example of this approach exposes clinicians passively to educational messages about the ‘costs’ of their decisions with no requirement to acknowledge or interact with the message.

Nudge-type interventions hold promise for improving health care delivery but their development and usage requires an evidence-base informed by appropriate research [14]. Evidence suggests adding brief cost feedback messages to blood test reports can reduce demand for the tests by NHS hospital doctors [19]. Research in the USA showed clinicians are aware of a responsibility to control costs, but in day-to-day decision-making they understandably prioritise the provision of the best available care to patients [20, 21]. It is unclear how NHS doctors in the UK perceive and respond to educational messages framed in terms of risk of harm to the patient and in terms of the financial cost to the health service.

**Fig. 1:**
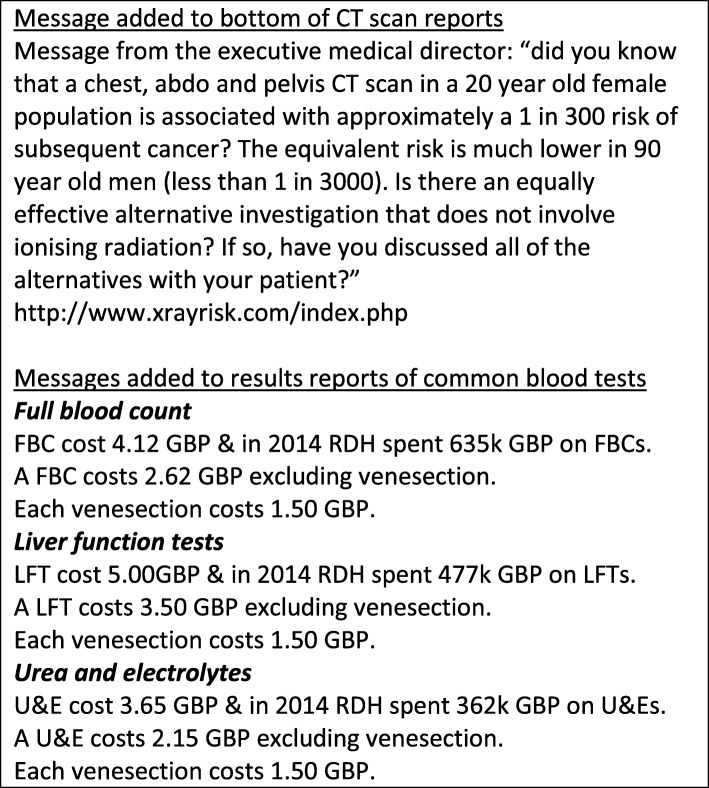
Intervention messages

We delivered a nudge-type intervention in a busy hospital in the UK from 1st February 2017 for a period of 12 months, which comprised of brief educational and informational messages added to all CT scan reports and common blood test results. The message on CT scan reports included information on the associated long-term health risks to the patient, whereas the message attached to blood test results contained financial cost information (Fig. 1). A controlled interrupted time series evaluation found a statistically significant reduction in one of the three blood tests targeted and in the number of CT scans performed during the intervention period [22, 23].

Qualitative methods have been used in the past to explore reasons why doctors order unnecessary tests and doctors’ response to related doctor-targeted interventions. Reasons identified for unnecessary preoperative tests included traditional practices, belief that other physicians want the tests done, medicolegal worries, concerns about surgical delays or cancellation, lack of training and lack of existence or awareness of evidence and guidelines [24, 25]. US physicians participating in focus groups identified the most common drivers of overuse to be patient expectations and lack of time to explain the risks [26]. In a qualitative evaluation of the Choosing Wisely Canada campaign, doctors reported that the intervention did not address several drivers of overuse they perceived as important: time pressures in the clinical encounter, uncertainty about the optimal care pathway and fear of litigation [27].

The present study aims to explore clinician attitudes towards our different nudge-type educational messages targeting overuse, why they did or did not influence decisions to order tests, how this depends on the patient and context, and factors that may impede or facilitate wider implementation and scaling up of the intervention.

## Methods

We conducted a qualitative study to address a need to understand attitudes and factors influencing doctors’ responses to the intervention and the drivers and barriers to wider adoption in the health service [28]. This involves a process of examining and interpreting data in order to generate meaning, develop understanding and improve knowledge, as opposed to testing theory.

Doctors at Royal Derby Hospital, UK, were invited to take part in a short telephone interview about their attitudes to the intervention and if/how it had affected their decision-making. They were invited initially via word of mouth by senior hospital clinicians involved in the study. In a later phase of recruitment an email was sent by the Medical Director of the NHS Trust to all doctors at the hospital inviting participation in the study. An information leaflet outlined the purpose of the study, explained that participation would be anonymous, the risks and benefits of taking part, and their right to withdraw at any time without reason. Interested individuals provided informed consent to participate by completing and digitally signing an online form. All participants were offered either a £20 gift voucher or a £20 donation to charity on their behalf. Interviews took place during the intervention period (no less than 9 months after the messages first appeared) and continued after the one-year intervention. Doctors expressing interest were telephoned and an interview arranged at a time convenient to them. Semi-structured telephone interviews were conducted using an interview guide designed around the aims of the research. Interviews were digitally audio recorded and transcribed verbatim. As the study formed part of an evaluation of health service delivery, ethical approval was not deemed necessary.

Transcripts were imported to NVivo™ qualitative analysis software and analysed using the Framework Method, a systematic and flexible approach to analysing qualitative data [29]. Analysis involved familiarisation with the transcripts, coding of the first five transcripts, development of a working analytical framework from these five transcripts, application of the framework to the remaining transcripts, charting the data into a framework matrix, then interpreting the data and generating themes by making connections within and between participants and coding categories in the matrix. An inductive and deductive approach was adopted in the coding to address the aims of the research whilst also allowing any unexpected themes to be identified. Author BY conducted the analysis, supervised by author JC who reviewed the working analytical framework and transcripts.

## Results

Twenty eight interviews were conducted, at which point no new themes were emerging from the interviews. Interview length ranged from 6.5 to 25 min (mean 10.5). Participants included a mix of junior and senior doctors from a range of specialties across the hospital (Table 1). A summary of the themes identified in the data explaining responses to each intervention message, along with exemplar quotes, is reported below.

**Table 1:** Participant characteristics

	n (%) [missing]
*Seniority*	
Foundation doctor	7 (25.0)
Training grade	4 (14.3)
Consultant	16 (57.1)
	[1]
*Medical specialty*	
Not applicable	8 (28.6)
Palliative care	4 (14.3)
Renal medicine	3 (10.7)
Surgery	3 (10.7)
Anaesthetics	2 (7.1)
Emergency medicine	2 (7.1)
Rheumatology	2 (7.1)
Critical care	1 (3.6)
Microbiology	1 (3.6)
Obstetrics and gynaecology	1 (3.6)
	[1]
*Gender*	
Female	18 (64.3)
Male	10 (35.7)
	[0]

### Response to cancer risk message on CT scan reports

The analysis generated six themes explaining responses to the cancer risk message: 1. Awareness of overuse and harm; 2. The message is inoffensive and harmless; 3. Increased confidence and disruptive impact; 4. Potential culture-changing tool; 5. Message not personally relevant; 6. Defensive medicine.

#### Theme 1: awareness of overuse and harm

Most doctors reported that they found the CT risk message to be highly acceptable. This was explained, firstly, by the message being consistent with their existing awareness of the need to prevent harm or overuse:

*I knew about it [cancer risk] but I also thought it was helpful because it does make you consider whether it [test] was necessary or not. (Consultant)*

*I think people are aware of it anyway but it is quite helpful to just put on there. (Foundation doctor)*

*A lot of requests for tests seems to be driven by clinicians because we have to have a diagnostic answer, not necessarily because the answer would influence management. [ … ] So when I saw it I was pleased that anything that makes people think twice about doing a scan. (Consultant)*

#### Theme 2: the message is inoffensive and harmless

High acceptability was explained, secondly, by the inoffensive and harmless way the message was delivered and the subconscious level at which it was perceived to operate:

*It’s not too irritating. Even though you’ve seen it so often it’s not irritating. (Grade unknown)*

*I think it’s a nudge. They’re all nudges. So people can take offence but it just registers in the subconscious almost. (Consultant)*

In contrast, a small number of doctors found the message distracting or thought it could cause more important information to be overlooked.

*I’d say if anything it just makes my decision-making more stressful because I get distracted at a point when I’m looking at quite critical information and trying to synthesise it. (Foundation doctor)*

*I think it’s really dangerous because it interrupts the clinical details and then there’s often a really important addendum added after the safety [cancer risk] message which gets missed. (Consultant)*

#### Theme 3: increased confidence and disruptive impact

Some doctors felt the message had impacted decision-making because it increased confidence.

*It’s really good to have extra reinforcement that sometimes not doing tests or doing different tests is just as important as going for the easier option. [...] But it’s really nice to see it written down for a lot of people because it gives them confidence. (Grade unknown)*

Other doctors stated that the message was simply a prompt that ‘made them think’.

*It’s something that we* should *think about but we don’t always think about necessarily, and I think it makes me question whether or not it’s actually required. (Training grade)*

#### Theme 4: potential culture-changing tool

Some doctors felt the intervention could increase awareness and contribute to culture change, including helping to make the public more aware of the risks.

*I think for the general culture of questioning so, juniors might not request one so much because they’re now aware there’s a danger to it. So I think it is changing sort of medical culture overall. (Consultant)*

*With patients who I’m not going to request something for, I think telling them about it anyway will hopefully change their expectations which will maybe change someone else’s practice in the future, because I know a lot of GPs get pressure about requesting things. (Training grade)*

#### Theme 5: message not personally relevant

Doctors reported that the message was not personally relevant to them and their practice, which was a theme overarching four sub-themes. Firstly, the information was already part of their clinical skills and decision-making:

*I do wonder if informed doctors actually need it, I mean I know I would think extremely hard about ordering up a scan willy-nilly because I’m aware of the radiation dosage involved so, maybe other doctors do but for me personally you’re sort of telling me what I already know. (Consultant)*

Secondly, doctors reported they were already more conservative than their colleagues at ordering tests.

*I hope I’m relatively conservative in what I order and I have been taking this into consideration. (Consultant)*

Thirdly, the risks were often less relevant to their medical specialty or patient group.

*It probably wouldn’t alter my behaviour because I work in palliative care and so most of my patients have already got cancer or a life-limiting deteriorating prognosis anyway. (Consultant)*

Finally, the message was seen as more relevant for doctors of a different seniority to themselves. Junior doctors reported the message was consistent with what they had learned in training but felt they did not have the authority to challenge the decisions of consultants:

*As a junior doctor when you’re just a minion you’re not the decision-making clinician. [...] But if this message was being seen by a decision-making clinician then that would make more of a difference. (Foundation doctor)*

Senior doctors felt the message would have been more effective at improving knowledge in junior doctors:

*I think that sometimes our junior doctors perhaps don’t appreciate [...] the radiation that some tests like the CT scan can deliver to a patient so I think it’s useful for them. Hopefully some of the senior doctors would know that already. (Consultant)*

#### Theme 6: defensive medicine

Defensive medicine refers to the fear of litigation influencing medical decision-making, including the overuse of tests and treatments. The theme of defensive medicine appeared highly influential in reports that the message had not influenced decision-making. This was described as a reluctance to ‘go against the guidelines’, or to take ‘unsupported risk’.

*I think we aren’t medicolegally supported in taking risks. [ … ] I do think there are some very strong forces out there in the world of medicolegal and the coroner and criticisms and the way the environment we now work in. (Consultant)*

### Response to financial cost message on blood test results

The analysis generated six themes explaining responses to the financial cost message: 1. Awareness of overuse and financial climate; 2. Improved my knowledge; 3. The message is not personally relevant; 4. Defensive medicine; 5. Patient factors are the priority; 6. The cost of a blood test is relatively small.

#### Theme 1: awareness of overuse and financial climate

The financial cost message was perceived as acceptable to doctors, explained by their awareness of the financial climate in which the NHS operates and the need to control costs.

*I think it’s reasonable to train people to realise there’s a cost to everything. (Consultant)*

*I completely agree with the concept that we do far too many blood tests without thinking about it. [...] We’re in a financially limited service and we’ve got to know the consequences of spending money on tests, so I think it’s information we must be given. (Consultant)*

#### Theme 2: improved my knowledge

In contrast to the cancer risk message, there was greater acknowledgment that the financial cost message had increased knowledge.

*I knew LFTs cost quite a lot of money. [ … ] I think yeah, it has given me a lot more information on it. (Foundation doctor)*

#### Themes 3 and 4: the message is not personally relevant; defensive medicine

Some cancer risk message themes were mirrored in the financial cost message data, including the perception that the message was not personally relevant and the need to practice defensive medicine:

*If [I] felt a test was necessary I’d do it regardless of cost but I hope that my test rate is much less than other doctors. (Consultant)*

*It’s a good idea but the amount of legal framework which is around the doctors [...] I don’t think, as a doctor, somebody would like to take a risk just because it costs a little more. (Foundation doctor)*

#### Theme 5: patient factors are the priority

However, doctors were more sceptical about whether the financial cost information had impacted behaviour. There was a theme that it was less appropriate to consider financial costs in decision-making and that patient factors were the priority.

*It is probably less appropriate [than cancer risk information] [...] As doctors looking after our patients on the ward on a day-to-day basis, we don’t make our decisions based on cost. We should only be making them on what’s the best thing for the patient at the particular time. (Foundation doctor)*

*I’m a bit ambivalent about it in some ways, I don’t think it’s bad to be thinking about it but I don’t think it should be the thing that drives our decision making, I think if a test is needed a test is needed. (Consultant)*

*Well it’s nothing to do with patient care, maybe overall in the scheme of the whole NHS but actually we have to look after individual patients. (Consultant)*

*It would be more relevant if it related to the patient, either anaemia or recurrent blood tests and harm and pain of having repeated needle sticks. (Consultant)*

#### Theme 6: the cost of a blood test is relatively small

Financial costs presented in the intervention message were sometimes perceived to be relatively trivial.

*I think it’s very difficult when some of the drugs that we know are out there that are being approved by NICE [The National Institute for Health and Care Excellence] are costing hundreds of thousands of pounds now [...] sometimes the small costs of the bloods feels like peanuts and I think it’s hard to influence people that way. (Consultant)*

*It will pale into insignificance compared to a stay in ICU for a week or a month for chemo, I know every little counts but I find the money side of it ... irrelevant? [...] It’s less relevant than the safety feature. (Consultant)*

### Evidence of impact on behaviour

The financial cost message appeared to have raised awareness in some doctors and increased their threshold for ordering blood tests.

*Before you’d sort of request a full set of bloods sort of not perhaps every day but maybe every other day, that sort of thing, but knowing the cost it’s made me think anyway maybe I’ll only request the one that I definitely, definitely need, rather than oh it would be nice to know this test, if I don’t think it’s clinically necessary it can wait another few days, then I will wait, so I think it’s influenced it in that way. (Foundation doctor)*

Another doctor reported that the financial cost message had been a major contributing factor in a wider-ranging change to blood test ordering that had since been implemented.

*Seeing the test results and the cost, I thought this was an unnecessary expense and through our service we have a high throughput of patients so, I worked out it could save the hospital five thousand pounds [...] so we’ve stopped doing routine full blood counts for pre-op, it’s an unnecessary test. (Consultant)*

There were patient and contextual factors highlighted as being important in whether or not the intervention messages affected decision-making. The information was felt to be especially relevant on repeat tests, helpful in informing patients of the risks of tests when they push to be tested, more influential when there is doubt about whether the test would be helpful, and when there are other imaging options available.

*In A and E, whenever it comes to a blood test you kind of have to do it, like it’s easy to know that you have to do it whereas I think maybe the blood tests thing [cost feedback] would make a bigger difference on the wards where you’re deciding how frequently do you repeat the blood tests for an inpatient. (Training grade)*

### Drivers and barriers to wider adoption of the intervention

A driver for wider adoption of the intervention was a demand from doctors to have more information about costs and numerical information about harm.

*So we personally have requested for us to have an idea of how much each test costs and our Trust hasn’t provided that yet. (Consultant)*

*I think most doctors like numbers, so if you give someone a ‘are you aware that this particular scan you requested has the same level of radiation as ‘X’ numbers of X-rays,’ it’s a useful reminder. (Training grade)*

It was also felt that a broader approach would be needed to achieve the required culture change to reduce unnecessary tests.

*For the culture change that’s required to think more carefully about what blood tests might be required, it’s going to be more than just about the cost of things but I think that is part of it, so I don’t know. It’s probably got to be part of a broader approach I would have thought. (Consultant)*

Several different suggestions were made to improve the effectiveness of intervention, including varying the message content, presenting the message at the point of ordering the test, distributing the information differently, e.g. at grand rounds, or from a different source, e.g. from a clinical colleague rather than senior management.

## Discussion

The findings indicate brief educational nudge-type messages targeting overuse of diagnostic tests by hospital doctors were highly acceptable to doctors. This was explained by themes of awareness of harm and overuse in health care and of the financial context in which the NHS operates. The nudge-type approach was generally seen as inoffensive and harmless although a small number of doctors were concerned about a potentially distracting effect of the message. Despite the controlled evaluation demonstrating the intervention was associated with a reduction in tests performed, the cancer risk message was not perceived by doctors to have impacted their decision-making because it was not personally relevant to them, for several key reasons. It improved interviewees’ confidence to question colleagues’ decisions and explain risks to patients and there was a perception it could have contributed to culture change. Doctors reported that the financial cost information was more likely than the cancer risk message to improve knowledge but it was not an important factor influencing decision-making. This was because patient-based factors were the priority and the cost of a blood test was relatively small. However, some examples were cited of change prompted by the financial cost message. In response to both messages, doctors cited the barriers of defensive medicine and a perceived lack of support in taking the risk of not performing a test.

The finding of positive attitudes to the intervention is consistent with evidence showing support for nudges provided they are perceived to be legitimately motivated and consistent with the interests and values of those targeted [14]. We observed an awareness in doctors of the motivations and drivers of the need to reduce harm and costs in health care, suggesting acceptability was high because the perceived values and motivations were shared by doctors. This is important because the indiscriminate nature of our approach could risk antagonising those whose behaviour is optimal. However, our study demonstrates a benefit of the nudge-type approach in that individuals do not feel personally targeted, which can prevent a defensive response to educational messages [30].

The trade-off resulting from this broad-brush method was that most doctors did not see the messages as personally relevant. This was because they felt they already considered the risks in their decision-making, were relatively conservative in their use of tests, the risk was less relevant to their practice or the message was less relevant to doctors of their seniority. Several studies have reported deficits in clinicians’ knowledge about the associated risks of CT scans [31, 32], suggesting there may have been an optimistic bias operating which should be addressed in future attempts to modify use of tests. Supplying clinicians with individualised feedback is an alternative strategy to address this finding, demonstrated to be effective in modifying prescribing but not test ordering, possibly because the latter is a more complex behaviour [33].

Defensive medicine was found to be a strong force in prohibiting doctors from taking what they perceived to be unsupported risks in reducing use of tests. This has been highlighted in previous qualitative research as a barrier to reducing unnecessary tests in the context of doctor-targeted interventions [24, 25, 27]. The finding that this is a key barrier to reducing use of tests in UK NHS doctors emphasises a need to improve perceived support in taking such risks.

Doctors acknowledged the cancer risk message made them think about the associated risks of CT scans and increased confidence to challenge decisions or discuss the risks with patients. They thought the messages could contribute to moving towards a culture of questioning and help to change the practice of unnecessary testing. This suggests that the intervention could have indirect effects on perceived norms that may benefit future strategies to target overuse of tests.

The financial cost message improved knowledge of these issues and we observed a demand from doctors for more information about costs. However, doctors were resistant to the idea that costs might influence decision-making and were dismissive about the relatively small cost of a blood test. This contrasts with evidence that more than two thirds of NHS doctors would change their practice if they were made more aware of the financial costs [34]. Despite some reported examples of change in practice in the routine use of blood tests, the findings suggested financial cost information might be perceived to be more effective as a supporting factor to patient-related information in attempts to influence the behaviour of NHS doctors.

Our previous controlled evaluation of the intervention found statistically significant reductions in demand for CT scans and full blood counts, a reduction in demand for urea and electrolytes of borderline significance, and no change in demand for liver function tests [22, 23]. There is a contrast between our quantitative and qualitative evidence about whether the intervention impacted decision-making. This may reasonably be expected when adopting a nudge-type approach targeting automatic processes in decision-making. Given that variations have been observed between doctors in test usage, it is also possible that behaviour change in a small number of individuals contributed disproportionately to change in test usage in the intervention hospital. For example, one participant reported implementing a change in their whole service as a result of the intervention. Additionally, the suggestion that the intervention message could have modified participants’ behaviour may have conflicted with self-identities we observed as either a junior doctor, and therefore more recently trained with the latest knowledge, or as a more experienced and knowledgeable senior doctor. The perception that other groups of colleagues will respond to the intervention may still have impacted individual behaviour in the absence of a conscious awareness of this effect.

This was a novel study of a population that can be difficult to engage in qualitative research. We demonstrated rigour by audio recording interviews, using an inductive and deductive method of analysis and the involvement of two researchers in the development of the coding framework. Interviews were conducted by non-clinical researchers who did not work at the intervention hospital and could take a neutral viewpoint on any issues arising. A limitation to this study is that doctors’ behaviour may have been influenced at a subconscious level through repeated passive exposure to educational messages. Participants may therefore have not been fully aware of how and why the intervention influenced their decision-making. Other potential methods for exploring clinician decision-making include observation, written/audio diaries, and recall and discussion of specific testing decisions made by the clinician. Hospital doctors may not have the time available to engage in such methods, however, and a short telephone interview may often be the most feasible application of qualitative methods to explore their behaviour.

There is no simple solution to improving professional practice in health care [35, 36] but our study showed a cheap and easy to implement approach to modifying demand for tests in a hospital setting was acceptable and holds potential for further development. The cancer risk message was reported to be more influential than financial cost information, although it had less perceived novelty, and both messages were not seen as personally relevant. The ways in which we found doctors responded to the intervention complements and aids understanding of our previously published findings that it was associated with reductions in demand for tests [22, 23]. There should, however, be modest expectations of such interventions and there is a need for high quality evaluation of nudge-type interventions in a health care setting, their behaviour change potential as part of a broader strategy to overcome the culture of defensive medicine, and their impact on inefficiency and harm.

## Conclusion

Our qualitative study demonstrates high acceptability of brief educational nudge-type messages that aimed to modify demand for CT scans and common blood tests by hospital doctors. Patient safety information was important in decision-making but did not represent new knowledge to doctors. Conversely, financial costs were new information that was unimportant in decision-making. This is a simple and cheap intervention that we found had prompted change in some individuals. However, doctors commonly reported that the messages were not personally relevant and their decision-making had not been impacted. A broader approach may be needed to address the overriding influence of defensive medicine identified in our study and to achieve greater efficiencies and harm reduction in health care. Our findings indicated doctors perceived this light-touch intervention can contribute to culture change and form a foundation for more comprehensive educational efforts.

## Data Availability

The dataset generated and analysed during the current study is not publicly available because personal narrative could identify individual participants and participants consented for data to be accessed only by the research team.

## Abbreviations

CT: Computed tomography
NHS: National health service
